# Bronchial epithelial cells of young and old mice directly regulate the differentiation of Th2 and Th17

**DOI:** 10.1042/BSR20181948

**Published:** 2019-02-01

**Authors:** Da Liu, Long He, Ning Ding, Wenjin Sun, Lulu Qiu, Li Xu, Aijun Jia, Cong Peng, Dongshan Zhang, Xudong Xiang

**Affiliations:** 1 The Second Xiangya Hospital of Central South University, Changsha, Hunan Province 410011, China; 2 Department of Respiratory Medicine, Changsha central hospital, Changsha, Hunan province 410111, China; 3 Department of Clinical Laboratory, Puai Hospital, Tongji Medical College, Huazhong University of Sciences and Technology, Wuhan, Hubei Province 430030, China; 4 Department of Emergency, Changsha Central Hospital, Changsha, Hunan Province 410004, China

**Keywords:** asthma, epithelial cells, Th2 cells, Th17 cells

## Abstract

To determine whether or not house dust mite (HDM) and HDM+lipopolysaccharide (LPS) exposure causes a difference in T-cell subsets from young and old mice. The bronchial epithelial cells (BECs) from young and old mice were divided into three groups (PBS (control), HDM, and HDM+LPS). CD4^+^ naive T cells from the spleen and lymph nodes were collected after 24 h of co-culture with BECs. The number of Th2 and Th17 cells was elevated in the HDM and HDM+LPS groups compared with the control group; these responses were exacerbated when exposed to HDM+LPS. The number of HDM- and HDM+LPS-specific Th2/Th17 cells in young mice was higher than old mice; however, the Th2:Th17 cell ratio was greater in young mice, whereas the Th17:Th2 cell ratio was greater in old mice. The expression of GATA-3 and RORc was increased in the HDM+LPS and HDM groups compared with the PBS group and exhibited most in HDM+LPS group. The expression of HDM+LPS-specific GATA-3 in young mice was higher, while the expression of HDM+LPS-specific RORc in old mice was higher. Murine BECs directly regulated CD4^+^ naive T-cell differentiation under allergen exposure.

## Introduction

Asthma is a chronic inflammatory disorder of the airways that is characterized by airway hyper-responsiveness and reversible airway obstruction [1]. Asthma occurs more frequently in the elderly (>65 years of age in Europe and the U.S.A. and >60 years of age in Asia) than usually expected [2]. The morbidity rate of acute asthmatic exacerbation reaches a maximum of 8–10% in early childhood, which decreases to nearly 5.5% in late adolescence, and increases to 8–10% in late adulthood [3]. Asthma in the elderly (AIE) is an important cause of morbidity and mortality [4]. Compared with adult asthma, airway obstruction is more apparent in the elderly, asthma control is poor, and the systemic inflammatory response and bronchial epithelial dysfunction are more severe [5]. There are at least two phenotypes of AIE, both of which have more severe airflow limitations and less complete reversibility in patients with long-standing asthma than patients with late-onset asthma [6].

The pathophysiologic mechanism underlying AIE is likely to differ from young asthmatics, which is positively correlated to age-related changes in respiratory and immunologic conditions. The increase in airway neutrophils with ageing has also been observed in asthmatic patients and the severe asthma phenotype is characterized by a predominantly neutrophilic airway inflammation [7,8].

The bronchial epithelium provides vital protection to the internal milieu of the lung from harmful agents by forming a physical barrier involving adhesive complexes and a chemical barrier consisting of mucus secretions [9]. The bronchial epithelium shows the capacity for self-renewal and clonal expansion during steady-state conditions and epithelial repair after injury [10]. Epithelial barrier dysfunction plays an important role in the asthma pathologic process [11]. The bronchial epithelium consists of cells that contact the environment and plays a pivotal role in immune surveillance by generating immune responses for appropriate activation of effector cells and antigen-presenting cells. There is increasing and solid evidence for the key role of bronchial epithelium tissues at the edge of environmental triggers in the process of the specific response balancing subsequent repair and inflammation. The impaired barrier effect may facilitate the passage of deleterious agents and/or antigens (DC capture or the efflux of mediators and inflammatory cells in the lumen) [12]. Derp1 is an antigen of house dust mite (HDM) derived from pollen, and is the most common allergen inducing asthma and the risk factors for persistent asthma in humans. Derp1 can not only activate epithelial cells to secrete a large amount of inflammatory medium, but can also damage the tight connection between cells, increase cell permeability, and consequently affect antigen-presenting cells [13,14].

Immune or inflammatory disorders are characterized by a critical imbalance in T-lymphocyte function; CD4^+^ naive T cells are essential for homeostasis. T cells appear to be strongly involved in allergic and inflammatory processes. Classically, the eosinophilic inflammation mediated by Th2 cells is considered to be a hallmark of asthma; however, nearly 10% of asthmatic patients also develop mixed inflammation consisting of eosinophils, neutrophils, and Th17 cells [15]. During the pathologic process of asthma, the inflammatory agents secreted by inflammatory cells can influence and stimulate bronchial epithelial cells (BECs) to secrete IL-8, CXC, and other neutrophilic chemotactic factors, inducing the recruitment and activation of neutrophils [16,17]. Th17 cells participate in airway neutrophil inflammation, and have been reported to have a relationship with severe, uncontrollable asthma that is not sensitive to glucocorticoids [18,19]. The increase in number of airway neutrophils with ageing has also been observed in patients with asthma, especially AIE. The elderly are prone to develop mixed asthma with neutrophil and eosinophil infiltration. Less is known about changes in Th17 cells caused by natural allergen exposure *in vitro* and the underlying immunologic mechanism is unclear. We assume that the number and proportion of Th17-to-Th2 cells will change when BECs are exposed to natural allergens; this change is different between elderly and young people.

Transcription factors, such as T-bet, GATA-3, and RORγt, are crucial for the differentiation from CD4^+^ naive T cells into Th1, Th2, and Th17 cells. GATA-3, a member of the GATA family of zinc-finger transcription factors, promotes Th2 differentiation, suppresses Th1 differentiation, directly up-regulates Th2 cytokine expression [20], and consequently enhances classic asthmatic responses. RORγt, a member of the nuclear receptor superfamily, was recently described as a master regulator for Th17 differentiation in the presence of TGF-β and IL-6 [21]. GATA-3 induces steroid-sensitive eosinophilic airway inflammation by enhancing the differentiation of Th2 cells and the production of Th2 cytokines, whereas RORγt induces steroid-insensitive neutrophilic airway inflammation by enhancing the differentiation of Th17 cells and the production of Th17 cytokines [22].

The aim of our study was to observe the function and correlation of BECs and T cells from young and old mice and further analyze the cellular basis and molecular mechanism underlying mixed asthma, which is characterized by activated Th17 cells in AIE.

## Materials and methods

### Mice

Wild-type (WT) C57BL/6 mice were purchased from the Animal Experiment Centre of Tongji Medical School. The male mice at 7–8 weeks and 13–14 months of age were used in all experiments. All animal studies were approved by the Institutional Review Board.

### BEC culture

Murine BECs were obtained by cold enzymatic digestion of murine bronchi or tracheas. Single cell suspensions from mice were cultured in 12-well plates that were coated with collagen I (50 µg/ml; BD Medical Technology, Franklin Lakes, New Jersey, U.S.A) at 3.5  ×  10^5^ cells/ml of MTEC proliferation media containing RPMI-1640 medium (Gibco-Thermo Fisher Scientific, Waltham, Massachusetts, U.S.A), 10% heat-inactivated FBS (Gibco-Thermo Fisher Scientific), retinoic acid stock B (10 mmol/l; Sigma–Aldrich, St. Louis, Missouri, U.S.A), insulin solution (6.25 mg/l; Sigma–Aldrich), epidermal growth factor solution (50 ng/ml; BD Medical Technology), bovine pituitary extract (25 mg/l; Sigma–Aldrich), transferrin solution (6.25 mg/l; Sigma–Aldrich), and cholera toxin solution (4.2 mg/l; Sigma–Aldrich). The submerged MTEC cultures were incubated at 37°C in a humidified incubator containing 95% air and 5% CO_2_. After 72 h, the supernatant and non-adherent cells were discarded. The adherent cells were allowed to differentiate for 10–14 days by replacing the proliferation medium with MTEC basal medium containing Nu-serum (2%; BD Medical Technology) and retinoic acid (10 mmol/l; Sigma–Aldrich).

### Immunofluorescence

BECs were adherent to chamber slides. Specimens were blocked in blocking buffer for 60 min. The blocking solution was aspirated and diluted anti-keratin antibody was applied (1:100; Abcam, Cambridge, Massachusetts, U.S.A) and incubated overnight at 4°C. The specimens were rinsed three times in 1× PBS (5 min each). The specimens were incubated in secondary antibody (1:50; Abcam) and maintained for 2 h at room temperature in the dark, then rinsed three times in 1× PBS (5 min each). The coverslipped slides were sealed using ProLong Gold Antifade Reagent with DAPI (5 μg/ml; Abcam).

### CD4^+^ naive T-cell isolation

Spleens from mice were collected and cells were purified from single-cell suspensions using a CD4^+^ naive T-cell isolation kit (Stemcell Technologies, Vancouver, British Columbia, Canada) according to the manufacturer’s guidelines. Following this, purified CD4^+^ naive T cells (2  ×  10^5^) were added to 12-well plates which had been added with RPMI-1640 medium containing soluble anti-CD3e (0.5 μg/ml; eBioscience, Waltham, Massachusetts, U.S.A), soluble anti-CD28 (1.0 μg/ml; eBioscience), and IL-2 (20 ng/ml; eBioscience). The cells were incubated with BECs for 24 h. Then, the cells were harvested for flow cytometry.

### BEC and CD4^+^ naive T cell co-culture

BECs were harvested when in good condition and irritated with 100 μg/ml of HDM (Indoor Biotechnologies, Charlottesville, Virginia, U.S.A), 100 μg/ml of HDM + 100 ng/ml of lipopolysaccharide (LPS) (Solarbio Life Sciences, Beijing, Beijing, China), or PBS for 8  h. The cells were then thoroughly washed with PBS. Splenic CD4^+^ naive T cells were collected. Purified CD4^+^ naive T cells were then cultured with BECs at a ratio of 10:1 (TCs:BECs). To analyze the subpopulation of T cells, the suspended cells were collected after 24  h and CD4, IL-4, and IL-17A concentrations determined by flow cytometry. The total protein of T cells was extracted for Western blotting.

### Flow cytometry

Cells were incubated with 100 ng/ml of PMA and 1 μg/ml of ionomycin for 1 h and 2 μM monensin for 5 h. The cells were collected and washed with buffer. The cells were incubated with diluted anti-CD4 FITC antibody (1:100; PeproTech, Rocky Hill, New Jersey, U.S.A) for 30 min at 4°C. The cell reaction was terminated with buffer and incubated with Cytofix/Cytoperm™ solution (BD Company, Franklin Lakes, New Jersey, U.S.A) for 20 min at 4°C. The cells were washed with 1× Perm/Wash™ solution (BD Company) and incubated with anti-IL4 PE (1:100; PeproTech) and anti-IL17A PE antibody (1:100; PeproTech) for 30 min at 4°C. The cells were washed twice with 1× Perm/Wash™ solution and analyzed. All flow cytometric data were acquired using a SORP LSRII Fortessa flow cytometer (BD Biosciences, Franklin Lakes, New Jersey, U.S.A).

### Western blotting

Equal amounts of protein were loaded into the wells of SDS/PAGE gels, along with molecular weight markers. Twenty micrograms of total protein were loaded from cell lysates. The gels were run for 1–2 h at 100 V. The proteins were transferred from the gels to the membranes for 2.5 h at 200 mA. The membranes were blocked for 1 h at room temperature, then incubated with appropriate dilutions of RORc (Proteintech Group, Rosemont, Illinois, U.S.A), GATA-3 (Proteintech Group), and GADPH antibodies (Proteintech Group) in blocking buffer at 4°C overnight. The membranes were thrice-washed in TBST (5 min each). The membranes were incubated with secondary antibody in blocking buffer at room temperature for 1 h. The membranes were thrice-washed in TBST (5 min each). Images were acquired using darkroom development techniques for chemiluminescence.

### Statistical analysis

Data obtained from experiments performed in triplicate and repeated at least three times are represented as mean ± S.E.M. Repeated-measure ANOVA was used to test the differences between specific T lymphocyte subpopulations and an unpaired Student’s *t* test was used to compare differences between two groups. *P*-values were considered significant at the 0.05 level.

## Results

### Number of Th2 and Th17 cells in the PBS, HDM, and HDM+LPS groups

**Figure 1:**
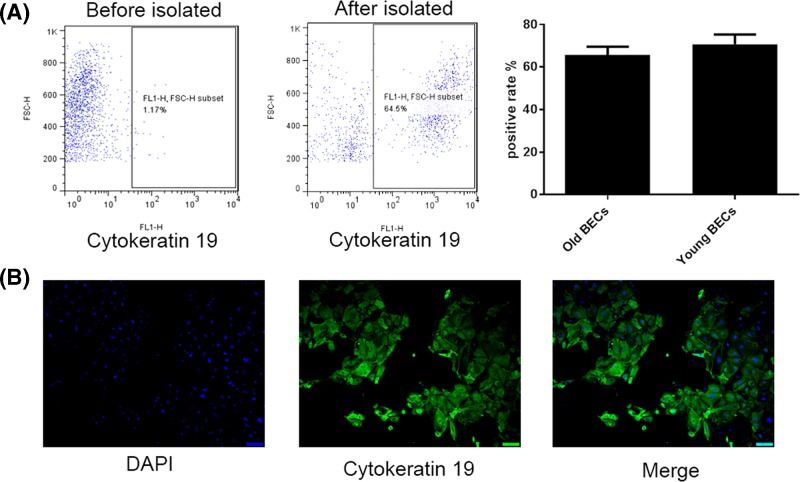
Culture of BECs *in vitro* on the seventh day The positive rate was assessed by flow cytometry and immunofluorescence using epithelial-specific cytokeratin 19 antibody. (**A**) The positive rate of primary culture of old BECs by flow cytometry. (**B**) The positive rate of primary culture of young BECs by immunofluorescence.

**Figure 2:**
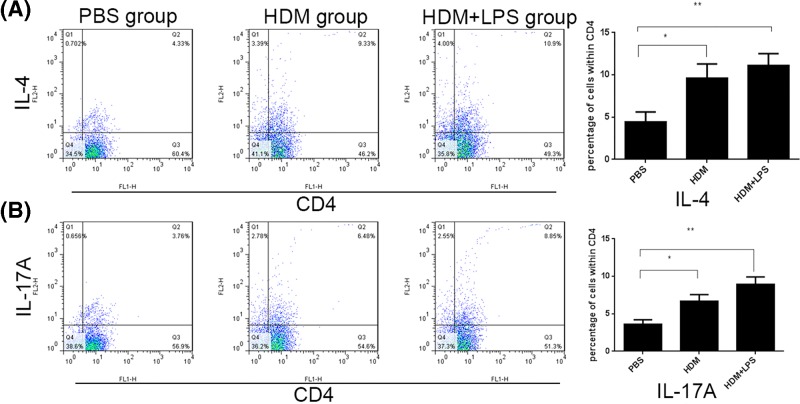
BECs from young mice regulate CD4^+^ naive T-cell differentiation *in vitro* (**A**) After irritation with 100 μg/ml of HDM, 100 μg/ml of HDM + 100 ng LPS, or PBS for 8  h and co-culture with CD4^+^ naive T cells for 24 h, the rate of Th2 cells increased in the HDM and HDM+LPS groups, but had the greatest increase in the HDM+LPS group. (**B**) After irritation with 100 μg/ml of HDM, 100 μg/ml of HDM + 100 ng/ml LPS, or PBS for 8  h and co-culture with CD4^+^ naive T cells for 24 h, the rate of Th17 cells increased in the HDM and HDM + LPS groups, but had the greatest increase in the HDM + LPS group (*, *P*<0.05; **, *P*<0.01).

After isolation, BECs from young mice had relative high cytokeratin 19 positive cell population rate than their counterpart from old mice as revealed by Flow cytometry (Figure 1A) and immunoflorescence (Figure 1B).The number of Th2 and Th17 cells were increased in the HDM-exposed and HDM+LPS-exposed groups compared in the control group, which was exposed to PBS. The number of Th2 and Th17 cells was higher in the HDM+LPS group than the HDM group (Figure 2A,B). These findings indicated that the immune responses may be regulated by BECs to differentiate naive T cells. In addition, the immune cells were enhanced when a higher degree of irritation was applied.

### Comparison of Th2 and Th17 cell number between young and old mice

**Figure 3:**
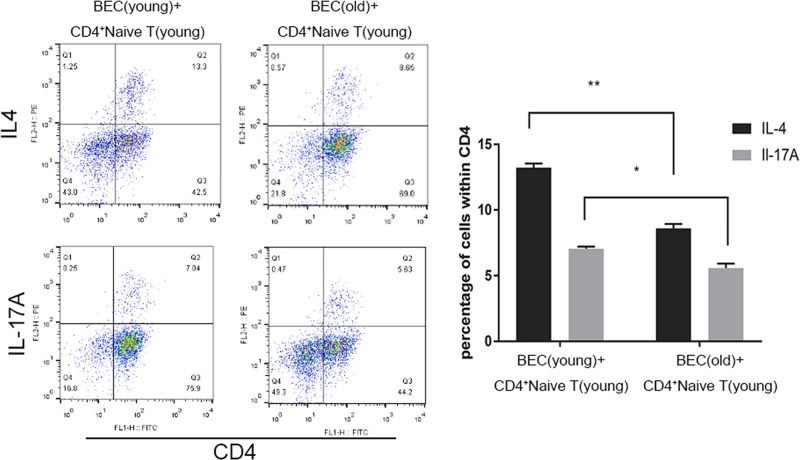
The number of Th2 and Th17 cells in young and old groups After 100 μg/ml of HDM + 100 ng/ml of LPS or PBS was given to BECs for 8 h, BECs and CD4^+^ naive T cells from young mice or BECs from old mice and CD4^+^ naive T cells from young mice were co-cultured for 24 h. T cells were collected and Th2 and Th17 cells were measured by flow cytometry (*, *P*<0.05; **, *P*<0.01).

BECs and CD4^+^ naive T cells from young mice, BECs from old mice, CD4^+^ naive T cells from young mice, and Th2 and Th17 cells were measured 24 h after HDM, HDM+LPS, or PBS exposure by flow cytometry. In each group, the number of Th2 and Th17 cells were increased after proper irritation compared within the PBS-exposed controls. The total proportion of Th2 and Th17 cells from young mice were higher than old mice (Figure 3). These results suggested that BECs from young mice may promote higher susceptibility to antigen and the immune response can be triggered more aggressively.

### Th2- and Th17-biased differentiation

**Figure 4:**
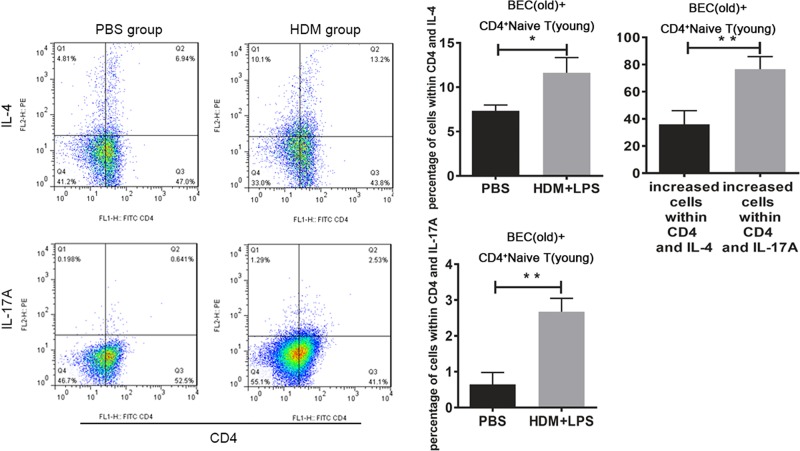
The number of Th2 and Th17 cells in the old group to HDM or HDM + LPS-specific exposure to BECs A total of 100 μg/ml of HDM + 100 ng/ml of LPS or PBS was given to BECs for 8 h. BECs from old mice and CD4^+^ naive T cells from young mice were co-cultured for 24 h. T cells were collected and Th2 and Th17 cells were measured by flow cytometry. The increased number of Th2 and Th17 cells were compared (*, *P*<0.05; **, *P*<0.01).

**Figure 5:**
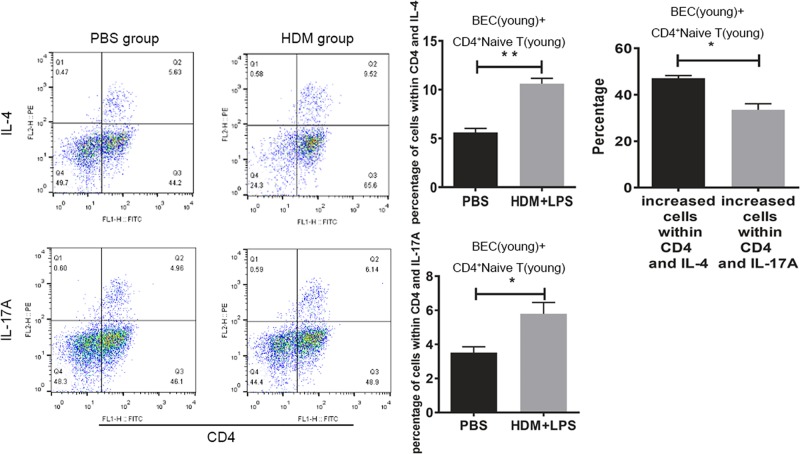
The number of Th2 and Th17 cell in the young group to HDM or HDM+LPS-specific exposure to BECs A total of 100 μg/ml of HDM + 100 ng/ml of LPS or PBS was given to BECs for 8 h. BECs and CD4^+^ naive T cells from young mice were co-cultured for 24 h. T cells were collected and Th2 and Th17 cells were measured by flow cytometry. The increased number of Th2 and Th17 cells were compared (*, *P*<0.05; **, *P*<0.01).

The number of Th2 and Th17 cells was determined in these two groups (BECs and naive T cells from young mice, BECs from old mice and CD4^+^ naive T cells from young mice). The number of Th2 cells was increased to a larger extent than Th17 cells, which indicated a Th2-biased differentiation with irritated BECs from young mice. In contrast, the number of Th17 cells was increased to a larger extent than Th2 cells, which indicated a Th17-biased differentiation with irritated BECs from old mice (Figures 4 and 5). These results are in agreement with the results of our previous study [23–25] obtained from an animal model *in vivo*.

### Expression of GATA-3 and RORγt

**Figure 6:**
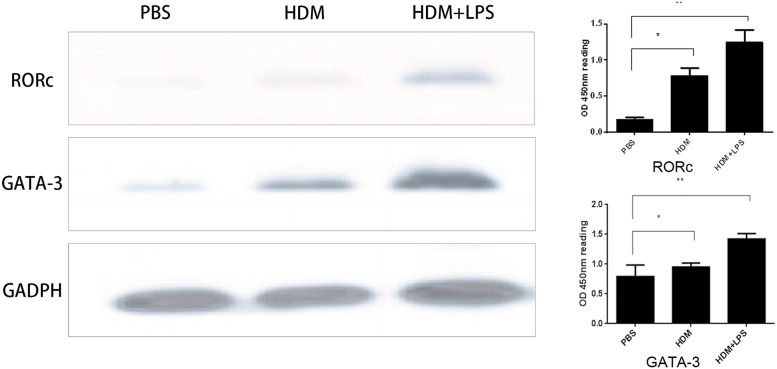
GATA-3 and RORc protein expression in HDM, HDM+LPS, and PBS groups GATA-3 and RORc protein expression in HDM, HDM+LPS, and PBS groups. A total of 100 μg/ml of HDM, 100 μg/ml of HDM + 100 ng/ml of LPS or PBS was given to BECs for 8 h. BECs and CD4^+^ naive T cells both from young mice were co-cultured for 24 h. T cells were collected and the total protein was extracted. The expression of GATA-3 and RORc was examined from the above groups by Western blotting. RORc and GATA-3 protein expression was normalized to GADPH (*, *P*<0.05; **, *P*<0.01).

**Figure 7:**
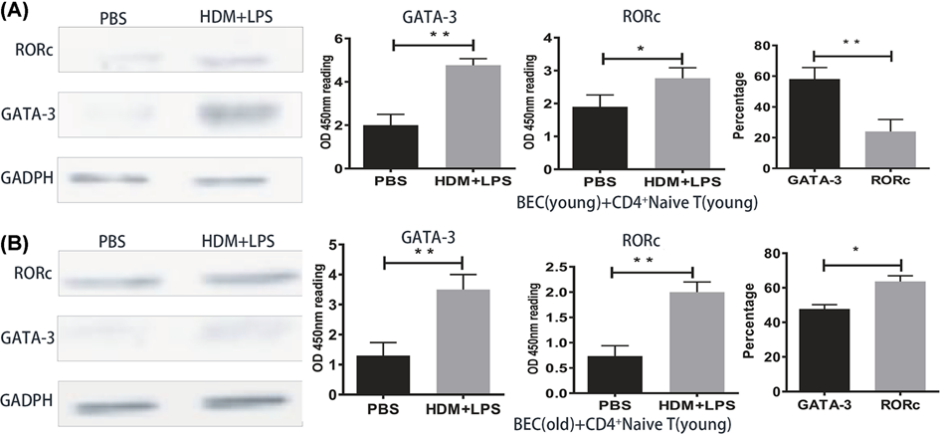
GATA-3 and RORc protein expression in young and old groups to HDM or HDM+LPS-specific exposure to BECs The young group contained BECs and CD4^+^ naive T cells from young mice (**A**) and the old group contained BECs from old mice and CD4^+^ naive T cells from young mice (**B**). A total of 100 μg/ml of HDM, 100 μg/ml of HDM + 100 ng/ml of LPS or PBS was given to BECs for 8 h. BECs and CD4^+^ naive T cells were co-cultured for 24 h. T cells were collected and the total protein was extracted. The expression of GATA-3 and RORc was examined from the above groups by Western blotting. RORc and GATA-3 protein expression were normalized to GADPH (*, *P*<0.05; **, *P*<0.01).

The levels of GATA-3 and RORc expression were increased in the HDM+LPS and HDM groups compared with the PBS group, especially in the HDM+LPS group (Figure 6). The degree of increased GATA-3 expression was larger than RORc when BECs were irritated by HDM+LPS from young mice (Figure 7A); however, the degree of increased RORc expression was larger than GATA-3 when BECs were irritated by HDM+LPS from old mice (Figure 7B).

## Discussion

Airway epithelial cells were previously thought to contribute to immune defense by forming a tight physical barrier toward the outside environment; however, a more complicated role of epithelial cells in immunity became evident recently based on the discovery of receptors of innate immune cells, such as TLRs [26,27], and several immune modulators, such as TSLP [28] and retinoic acid [29], that are expressed in or secreted by these cells for direct and indirect cell contact with local professional immune cells, further adjusting the level of reactivity of classical immune cells [30,31]. Thus, epithelium may ascribe a potential immune function to the epithelial barrier. The specific role epithelium plays and which part epithelium reacts to in developing the immune response remains unknown.

It has also been shown that HDM-induced asthma leads to significant injury to lung tissue, especially the bronchial epithelium [32]. LPS acts as the prototypical endotoxin by activating a number of transcription factors. With respect to the level of exposure at very low levels (<10 ng), LPS induces suppression [33], while at levels of approximately 100 ng, LPS promotes Th2-mediated inflammation due to adjuvant effects [34]. Our observations revealed that sensitization with 100 μg/ml of HDM and 100 μg/ml of HDM + 100 ng/ml of LPS enhanced Th2- and Th17-type responses *in vitro* under the control of BECs. BECs could serve as a kind of antigen-presenting cell to regulate the differentiation of T cells. This finding underlies the immune role and critical part of immunologic diseases, such as asthma, by definition of epithelial disease.

The elderly are prone to suffering from severe asthma more easily than young patients [6,35]. We previously established an asthmatic animal model, analyzed the cell population of bronchoalveolar lavage fluid, and observed the pathologic changes in lung tissue. Young mice presented a Th2 response with eosinophilic infiltration, but the Th17 response occurred in old mice with an eosinophil–neutrophil mixed infiltration. In the present study, it was observed that irritated young BECs triggered a more apparent differentiation of T cells than old BECs, which may have dependence in age-related cellular status and physiologic function. Irritated BECs from young mice induced significant Th2 responses upon exposure to HDM+LPS. Indeed, the irritated BECs from old mice induced Th17 cells to increase in number more than Th2 cells. This finding was in agreement with the asthma animal model, as previously described [23–25]. Thus, the immune responses of the young group were more apparent than the old group, but the differentiation directions were not the same. Moreover, the immune responses were enhanced, which was in agreement with a higher degree of immunologic stimulation, such as the addition of LPS.

The current study demonstrated that the phenotype of asthmatic airway inflammation is determined by T cell-specific transcription factors. GATA-3 induced steroid-sensitive eosinophilic airway inflammation by enhancing Th2 cell differentiation and Th2 cytokine production, whereas RORγt induced steroid-insensitive neutrophilic airway inflammation by enhancing Th17 cell differentiation and Th17 cytokine production. The up-regulation of GATA-3 [36,37] has been demonstrated clinically in T cells from the airways and peripheral blood of asthmatics. Transcription factors also have a crucial role in the development of airway remodeling by mediating Th1/Th2 cell differentiation [38]. GATA-3 induces Th2 cell development by promoting Th2 cytokine expression, but simultaneously inhibits Th1 differentiation by inhibiting T-bet, and thus plays a critical role in asthma [39,40]. The precise role of RORγt in asthma has not been fully clarified, but it has been reported that expression of RORγt or RORc, which encodes RORγt, is increased in the peripheral blood mononuclear cells of asthmatics [41,42]. Interestingly, neutrophils predominantly infiltrate the airways of RORγt-overexpressing mice, with enhanced expression of IL-17A and IL-22 in the lungs after exposure to the same antigen [22].

Therefore, it appears that an increased level of RORγt may be implicated in the mechanism underlying severe asthma. We observed that the expression of GATA-3 and RORc of HDM and HDM+LPS groups was increased compared with the control group, and there was higher expression of GATA-3 than RORc in the young groups. In contrast, the expression of RORc in old groups was higher than GATA-3. These results revealed that GATA-3 and RORc may be a major molecular mechanism of enhancing eosinophilic and neutrophilic airway inflammation by the stimulator under Th2- and Th17-biased conditions.

In conclusion, asthma is a heterogeneous disease with different phenotypes and variable clinical manifestations which depend on the age, gender, genetic background, and environmental influences on the patients. Our study provides an important step to clarify the intricate pathogenesis of elderly or severe asthma. Furthermore, the epithelium may be the target of a novel strategy for the treatment of asthmatic patients.

## Abbreviations

AIE: asthma in the elderly
BEC: bronchial epithelial cell
CD-4: cluster of differentiation-4
CXC: C-X-C chemokine
GADPH: glyceraldehyde-3-phosphate dehydrogenase
GATA-3: GATA Binding Protein 3
HDM: house dust mite
IL: Interleukin
LPS: lipopolysaccharide
MTEC: Medullary Thymic Epithelial Cell
ROR: Retinoid-Related Orphan Receptor
RPMI-1640: Roswell Park Memorial Institute-1640
TBST: Tris-buffered saline and Tween 20
Th: T-helper
TLR: Toll-like receptor
TSLP: Thymic stromal lymphopoietin
